# What can we learn from trial decliners about improving recruitment? Qualitative study

**DOI:** 10.1186/s13063-016-1626-4

**Published:** 2016-10-12

**Authors:** Adwoa Hughes-Morley, Bridget Young, Roelie J. Hempel, Ian T. Russell, Waquas Waheed, Peter Bower

**Affiliations:** 1 MRC North West Hub for Trials Methodology Research, Manchester Academic Health Science Centre, University of Manchester, Oxford Road, Manchester, M13 9PT UK; 2 York Trials Unit, Department of Health Sciences, University of York, York, YO10 5DD UK; 3 MRC North West Hub for Trials Methodology Research, Department of Psychology, Institute of Psychology, Health and Society, University of Liverpool, Liverpool, UK; 4 School of Psychology, University of Southampton, Southampton, SO17 1BJ UK; 5 Swansea University Medical School, Swansea University, Swansea, SA2 8PP UK; 6 NIHR School for Primary Care Research, Manchester Academic Health Science Centre, The University of Manchester, Williamson Building, Manchester, M13 9PT UK

**Keywords:** Randomised controlled trials, Non-participation, Depression, Qualitative research, Recruitment

## Abstract

**Background:**

Trials increasingly experience problems in recruiting participants. Understanding the causes of poor recruitment is critical to developing solutions. We interviewed people who had declined a trial of an innovative psychological therapy for depression (REFRAMED) about their response to the trial invitation, in order to understand their decision and identify ways to improve recruitment.

**Methods:**

Of 214 people who declined the trial, 35 (16 %) gave permission to be contacted about a qualitative study to explore their decision. Analysis of transcripts of semi-structured interviews was informed by grounded theory.

**Results:**

We interviewed 20 informants: 14 women and six men, aged 18 to 77 years. Many interviewees had prior experience of research participation and positive views of the trial. Interviewees’ decision making resembled a four-stage sequential process; in each stage they either decided not to participate in the trial or progressed to the next stage. In stage 1, interviewees assessed the invitation in the context of their experiences and attitudes; we term those who opted out at this stage ‘prior decliners’ as they had an established position of declining trials. In stage 2, interviewees assessed their own eligibility; those who judged themselves ineligible and opted out at this stage are termed ‘self-excluders’. In stage 3, interviewees assessed their need for the trial therapy and potential to benefit; we term those who decided they did not need the trial therapy and opted out at this stage ‘treatment decliners’. In stage 4, interviewees deliberated the benefits and costs of trial participation; those who opted out after judging that disadvantages outweighed advantages are termed ‘trial decliners’. Across all stages, most individuals declined because they judged themselves ineligible or not in need of the trial therapy. While ‘prior decliners’ are unlikely to respond to any trial recruitment initiative, the factors leading others to decline are amenable to amelioration as they do not arise from a rejection of trials or a personal stance.

**Conclusions:**

To improve recruitment in similar trials, the most successful interventions are likely to address patients’ assessments of their eligibility and their potential to benefit from the trial treatment, rather than reducing trial burden.

**Trial registration:**

International Standard Randomised Controlled Trial Number: ISRCTN85784627. Registration date 10 August 2011.

**Electronic supplementary material:**

The online version of this article (doi:10.1186/s13063-016-1626-4) contains supplementary material, which is available to authorized users.

## Background

Randomised trials are strongly recommended for evaluating interventions, yet recruitment of participants is an increasing problem [1–3]. In developed countries, there have been considerable efforts to improve recruitment through legislation and infrastructure [4–6]. Recent reports in the United Kingdom (UK) suggest that more people than ever are being approached to participate in trials [6]; however the proportion of people who enrol is small and recruitment remains a problem, with between 45 % and 80 % of trials failing to meet recruitment targets [2, 7]. The difficulties may be even more pronounced when enrolling patients with depression, with many examples of trial failure due to poor recruitment [8, 9]. The challenges stem from sources including: the stigma of mental illness; poor identification of mental disorders by clinicians; diagnoses which adversely affect patients’ ability and motivation to participate in research; and mistrust [10, 11]. Consequences of poor recruitment include increased costs, reduction in statistical power and continued use of interventions that are ineffective or harmful to patients [12, 13].

There is a dearth of evidence-based interventions for improving recruitment into trials, leading to calls for the development of ‘*a science of recruitment*’ [1, 14]. Recruitment is now a methodological research priority for trials units in the UK [15], and systematic reviews have identified an urgent need for robustly evaluated interventions, particularly those tested in the real world [16, 17].

The Medical Research Council (MRC) Complex Interventions Framework provides a useful basis for developing and evaluating interventions to improve recruitment [18, 19]. Qualitative research has an important role to play in the development of interventions [20–22]. To improve recruitment, it is important that this development work is informed by the perspectives of people who decline trials. However, our meta-synthesis of factors affecting recruitment into depression trials [23] found that only one of the 15 studies included decliners [24]. The remaining studies all focused on the perspectives of staff, or of patients successfully recruited. Furthermore, all of the studies focused on respondents’ reported reasons for their decision, but did not explore in detail their accounts of what happened when they received the invitation to join a trial. This may have elicited idealised justifications and failed to take into account *deliberation,* an important aspect of decision making identified by the ‘deliberation and determination’ framework [25, 26]. Understanding responses to the invitation to join a trial and how the decision to decline is reached may assist trialists to enhance recruitment by designing interventions to address shortcomings. By exploring this important gap in our understanding, we aimed to shed light on what has been termed a ‘blind spot in the literature’ on recruitment [27].

We therefore explored interviewees’ accounts of what they did and what happened when they received the trial invitation. Rather than simply asking for reasons why they declined trial participation, which might elicit idealised justifications rather than deliberations and reasons, we explored informants’ accounts of *how* they reached their decisions and the factors that affected them.

## Methods

### Setting: the REFRAMED trial

This qualitative study explored interviewees’ responses to receiving an invitation to participate in the REFRAMED trial (REFRActory depression - Mechanisms and Efficacy of Dialectical Behaviour Therapy) [28]. REFRAMED evaluated the effectiveness of Radically Open Dialectical Behavioural Therapy (RO-DBT) [29] for treatment-resistant depression. It recruited trial participants through general practices and mental health services in Dorset and Hampshire in England and Gwynedd in North Wales. Those eligible were: aged over 18 years; had a current diagnosis of depression; and had not responded to antidepressants. All invited individuals received a ‘summary participant information leaflet’ (Additional file 1) and those who were interested took part in full eligibility assessments. Eligible individuals who consented were randomised to RO-DBT in addition to usual care and antidepressant medication, or to usual care and antidepressant medication. RO-DBT comprised 29 weekly individual therapy sessions lasting 50 minutes and 27 group skills sessions lasting 2.5 hours. While some components of RO-DBT are common to all behaviour therapies, RO-DBT uniquely targets social-signalling deficits, focuses on changing internal experience (for example emotion dysregulation, cognitive distortions and traumatic memories) and also teaches clients how to express emotions appropriate to context and use non-verbal social-signalling strategies known to enhance social connectedness. REFRAMED participants were assessed four times over 18 months – at baseline and after 7, 12 and 18 months; in addition RO-DBT participants completed monthly questionnaires over 18 months.

### Qualitative study

The qualitative study was informed by an epistemological standpoint of pragmatism, a perspective that embraces methodological pluralism and is increasingly used in health services research to inform the development and evaluation of interventions that are transferable and usable in real life [30, 31]. Pragmatism focuses on ‘what works’ and on generating solutions to existing problems by identifying and integrating effective strategies to build on the strengths and reduce the inherent flaws of each [32, 33]. Our pragmatic approach enabled us to use different methods of sampling, data collection and analysis to address our research aims, including techniques from grounded theory [34]. Grounded theory aims to generate theories of social phenomena grounded in systematic analysis of data and is particularly appropriate for explaining social processes. We offered individuals who had declined the REFRAMED trial the choice between being interviewed by telephone or email. These options were informed by: advice from two patient and carer engagement groups – the UK Clinical Research Network Mental Health Service User Research Panel (SURP) and Primary care Research In Manchester Engagement Resource (PRIMER); advice from trialists who had worked with similar groups; literature suggesting that decliners would be reluctant to take part in face-to-face interviews [24]; and evidence that well-planned telephone and email interviews can gather the same data as interviews face to face [24, 35] and promote access to ‘isolated, geographically dispersed or stigmatised groups who are often overlooked or ignored’ [36, 37].

### Sampling and recruitment

Most of the 1867 patients approached for REFRAMED were identified from electronic health records in general practices and community mental health teams by searching for patients diagnosed with depression who were receiving repeat prescriptions of antidepressants. Of the rest, a few referred themselves, but most were referred to REFRAMED by their general practitioners (GPs), care coordinators and psychiatrists. We could not access those who declined their clinicians’ invitations so our sampling for the qualitative study focused on the 214 patients who responded to postal invitations from general practices and community mental health teams by returning reply slips to decline REFRAMED, in particular the 35 who expressed interest in participating in the interviews and provided contact details.

We initially sampled 12 interviewees for maximum variation [38] in the following characteristics: age, gender and geographic location. In line with the principles of grounded theory, we then sampled theoretically [39], using information provided on decliners’ reply slips. We invited eight interviewees who gave different reasons for declining and who we therefore felt were ‘deviant’ or could provide accounts that would help us to develop our analyses further [34]. We continued sampling until we achieved data saturation; that is until no new themes emerged.

### Data collection

One of us, AH-M, a health services researcher undertaking a PhD with training in qualitative interviewing, contacted those who expressed interest – by telephone or email according to their preferences – to discuss the qualitative study. Having had no prior contact with interviewees, she explained that she was linked to the REFRAMED team but independent of both them and patients’ clinical teams, and sought consent from potential interviewees. Arrangements were made to conduct telephone or email interviews at a later date with those who consented. Audio interviews were recorded and professionally transcribed in an ‘efficient verbatim’ style, that is by transcribing content but not pauses or hesitations. AH-M checked transcripts for accuracy and pseudonymised them.

Recruitment to REFRAMED took place between March 2012 and May 2015 and the qualitative interviews took place between August 2013 and January 2015 – within 3 months of interviewees declining to participate in REFRAMED so as to minimise recall bias. To allow full exploration of topics, interviews were conversational and responsive to participants. To ensure consistency across interviews, questions followed a topic guide (Additional file 2), which was piloted and based on relevant literature and consultation with SURP and PRIMER, our patient and carer engagement groups. Interviews initially explored participants’ recollection of and thoughts about: being invited into the trial; making the decision to decline; understanding the research and trial interventions; and talking therapies, in particular RO-DBT. Interviews focused on the period when respondents first received the invitation into the REFRAMED trial, and asked them to describe in detail what they did, who they talked to, and what they thought. We made field notes during interviews and modified the topic guide in response to early interviews. To minimise interviewee burden, transcripts were not returned to respondents, nor were they asked to provide feedback on findings.

### Data analysis

Analysis was interpretive and drew on constant comparison with grounded theory [34]. The iterative analysis process was led by AH-M who read and reread transcripts to develop preliminary codes to identify themes and theoretical categories [40], which we gradually developed into a conceptual framework. Coding was combined with a holistic consideration of transcripts to retain the context of participants’ accounts and identify and interpret aspects that participants were silent about or did not emphasise relative to the accounts of other participants, or which did not fit the rest of their account. In discussion with BY and PB, AH-M continually reviewed emerging themes and categories in the light of new data, modifying these to ensure they fitted the data whilst accounting for deviations. Some categories and themes arose from inductive analysis, while others drew more deductively on literature from our systematic review [23]. This flow from data to literature, and back to the data, refined the codes and the developing theoretical constructs [41]. The multi-disciplinary team developed the analysis and ensured its ‘trustworthiness’ [42, 43] in a process of investigator triangulation. Analysis was assisted by NVivo 10.

To illustrate our interpretations we include selected quotations from our data. These are broadly representative of the key themes, whilst also reflecting a range of views. Quotation labels indicate participants’ age, gender, identification number and stage at which they declined; for example ‘67F01S03’ indicates a 67-year-old female who was our first participant and declined at stage 3. Text within square brackets [] indicates clarifications that we have inserted; ellipses ‘…’ indicate pauses by respondents; and ellipses within square brackets […] indicate omitted text.

## Results

### Participant characteristics

Of the 35 patients initially expressing interest, two declined to be interviewed when contacted and eight did not respond to our attempts to contact them. The remaining five were not interviewed as we had reached theoretical saturation. We undertook 20 interviews with 14 females and six males – 18 by telephone, one by email and one by both telephone and email. Apart from the interviewee and the researcher, no other persons were present during the interviews. Telephone interviews lasted between 16 and 76 minutes with a mean of 30 minutes. The email interview took place over the course of one week; and for the combined interview the telephone interview occurred first, followed by one day’s email correspondence. The mean age of the 20 who participated in the qualitative study was 57 years; the mean age of the 252 who participated in REFRAMED was 45 years and that of the 214 who declined was 50 years. Of the 20 interviewees, 18 described themselves as ‘white British’, one as ‘white other’ and another as ‘Asian British’. Ten were retired, six were unemployed, three were employed full time and one was a full-time student. Ten interviewees had prior experience of being invited to participate in a trial. Table 1 lists the characteristics of interviewees.

**Table 1:** Characteristics of study participants

Participant number	Age	Site	Gender	Highest educational qualification
1	67	England	F	Secondary school
2	18	Wales	F	Secondary school
3	67	Wales	M	Secondary school
4	54	England	F	University degree or higher
5	74	England	F	Secondary school
6	59	England	M	Secondary school
7	62	Wales	M	University degree or higher
8	70	England	F	Secondary school
9	44	England	F	Secondary school
10	73	England	F	Secondary school
11	77	England	F	University degree or higher
12	66	Wales	M	University degree or higher
13	63	England	M	Secondary school
14	69	England	F	Secondary school
15	40	England	F	University degree or higher
16	46	Wales	F	University degree or higher
17	61	England	F	Secondary school
18	50	England	F	Secondary school
19	38	England	M	University degree or higher
20	45	Wales	F	University degree or higher

### Overview of informants’ decision making

Ten interviewees read the trial invitation with experience of having made trial participation decisions in the past. Our analysis of their accounts of their response to receiving the trial invitation indicated that they passed through up to four sequential stages in making the participation decision: (1) assessing the nature of the invitation; (2) assessing their own eligibility; (3) assessing their own need for trial therapy and potential to benefit; and (4) comparing the risks with the rewards of participation. While all informants engaged in stage 1, two described opting out of the trial at this stage without further deliberation. Of those progressing to stage 2, nine declined at this stage, seven at stage 3, and two progressed to stage 4 before finally declining. Thus while two progressed through all four stages of this process, the majority reached their decision earlier. However, the content of informants’ deliberations did not always reflect this sequential order, for example some considered the potential to benefit from the therapy (stage 3) before assessing their eligibility (stage 2). In reporting their accounts, we characterise different ‘types’ of decision makers to distinguish the decisions that interviewees made at each stage of the process of responding to the REFRAMED invitation.

#### Stage 1: assessing the nature of the invitation

In the REFRAMED trial GPs and mental health teams sent invitation letters to potential participants without prior notice. Informants generally reported opening the letter without delay and reading it with the trial response form. Some reported that they briefly glanced through the accompanying REFRAMED summary leaflet or did not read it, while others described reading the leaflet in detail. With one exception, informants reported that: they approved of being sent the trial invitation; the letter format was appropriate; and being invited in this way was good because it enabled them to make decisions in their own time:

*‘The letter is a good idea…I mean if they sign you up you have to decide very quickly and you don’t have time to chew over the information, so having a letter makes sense, you can sit and think about it and decide what to do’.* (66M12S2)

The exception was an interviewee widowed one year before receiving the trial invitation. She reported that, given her personal circumstances, she would have expected her GP to have removed her name from the list of patients to be sent the invitation. However, she acknowledged that for people experiencing ‘normal depression’, being sent such an invitation was not only appropriate, but would actually be positive:

*‘It probably is a good thing really, if I’m honest. I mean, it’s the only way you get to know things, isn’t it?… Like, say, I’d got some illness, I suppose it’s the only way you’re going to find out things isn’t it, what tablets I’m on, whether they work and all that sort of thing. I think perhaps if I’d been depressed normally, like, I mean, a lot of people are, aren’t they, and they’re on depression tablets for a while. I can understand that’.* (70F08S2)

The other interviewees expressed positive views about research and the trial specifically, particularly the need to improve health services and advance knowledge through such endeavours:

*‘Without research no-one would ever get anywhere, would they? So even if it didn’t help me, it would still help, you know, others wouldn’t it?’* (44F09S3)

Many respondents reported that they supported REFRAMED’s aim to evaluate a new treatment for depression, and were comforted to know *‘that somebody was doing something about it’* (67F01S3).

For ten informants this was not the first time they had been invited by letter to participate in research. Of these, eight reported having accepted at least one invitation. Three of these were trials of psychological therapy for depression; one a psychological experiment including mood assessment; two studied bowel cancer; one respiratory illness; and one vision. Being sent such letters was seen as a necessary part of the research process, regardless of whether the invitation was declined or accepted. Crucially, interviewees felt able to make whatever decision felt right for them, including declining, so did not mind being invited:

*‘I didn’t mind actually because I know that the [general practice] was very into research and I believe that the surgery itself was one of the best in the country for research. I had been sent them on, I think, about bowel cancer and, I can’t remember, two or three other things and I must admit that my reaction was just the same’.* (74F05S1)

This interviewee, whom we categorised as a ‘prior decliner’, reported that REFRAMED was one of several trials that she had declined owing to concerns about confidentiality. The other ‘prior decliner’, who reported having declined all invitations, was the oldest of our interviewees, and cited her advanced age as the reason for not accepting trial invitations:

*‘I’m 77…when you get to this age, you realise that you just take every day at a time, and I don’t want anything that I haven’t got to have, because I’ve had two hip replacements, I’ve had an operation on my back, and to be quite honest, as I say, I don’t want anything that isn’t necessary. I don’t think that at this stage in my life, [trials] apply to me, really’.* (77F11S1)

Thus these two ‘prior decliners’ had made prior decisions not to participate in trials for different reasons – confidentiality and being ‘too old’. Yet both accounts centred on their personal circumstances and their policy of declining all trial invitations, and both declined very quickly and with little deliberation, as they had established a precedent.

In contrast most interviewees reported making decisions that took account of the features of each trial presented to them. The remaining 18 interviewees, including eight who had previously participated in research, made decisions specific to the REFRAMED trial. These interviewees approached the REFRAMED decision with positive attitudes despite perceiving mixed outcomes from that previous research:

*‘It was excellent… And it’s been the greatest help I’ve ever had actually. I mean, 40 years I’ve been suffering with depression but this came at a latter stage of my life obviously and I took it’.* (62M07S3)

Whilst others found it to be of less direct benefit:

*‘I think the person that was doing [the study] got more benefit than I did. I was just helping that person out, which I didn’t mind doing’.* (54F04S2)

Interviewees with no experience of trials often recounted experiences of close family members who had made decisions to enrol in trials, described supporting their family members’ decisions, and displayed positive opinions and detailed knowledge of those trials:

*‘One of my husband’s problems is that he now has end-stage kidney failure, and has had for the last 8 years. When he was initially diagnosed with chronic renal failure [pharmaceutical company] were instigating a massive worldwide research into statins and the effect on renal failure. My husband agreed to enter into that and I appreciate that they basically give people either a placebo or the real drug[…]And it was perfectly obvious from my husband’s statins – prior to taking the drug, his cholesterol was six-something and 3 months after it had gone down to two. So it was pretty obvious that he didn’t have the placebo.’* (74F05S1)

Thus interviewees were universally positive about the trial, even the ‘prior decliners’.

#### Stage 2: determining own eligibility

With the exception of prior decliners, all respondents described engaging with trial eligibility on reading the letter. Interviewees described: the trial eligibility criteria; their perceptions of their eligibility for the trial; and their identification by clinical teams who sent them the trial invitation. Their accounts revealed differences in the interpretation of the diagnosis and management of depression. Nine interviewees described using the trial information and eligibility criteria to decide how to respond to the invitation in light of their personal circumstances. They fell into two broad ‘self-excluding’ categories: those who judged that they were ineligible because they were not taking antidepressants prescribed by their clinical teams (though they may have been considered eligible by those teams); and those who described themselves as ‘not depressed enough’.

The trial eligibility criteria required patients to have a current diagnosis of major depressive disorder, to have been prescribed antidepressants, and not to have responded to these within the current episode. All participants in this qualitative study had been identified by their clinical teams as matching these criteria. Six interviewees reported that, when invited into REFRAMED, they had never taken their prescribed antidepressants, or soon stopped doing so, without informing their doctors. They had decided not to participate in the trial, perceiving that they were ineligible for the trial, rather than rejecting the trial itself. One reported that doctors had prescribed him antidepressants on several occasions, but he had always refused to take them, because he felt strongly that he did not need them to manage his mood, and worried about the effects of long-term antidepressant use on his health:

*‘I really do believe going onto antidepressants, particularly long-term, is not a good thing’*. (62M07S3)

Other interviewees, who had initially taken their prescribed antidepressants, reported that they had stopped taking them without consulting their doctors when they felt they no longer needed medication, or they did not *‘like taking them’* (59M06S2). Several interviewees reported side effects from the antidepressants, which they had managed by stopping their medication. Another interviewee described asking her GP to stop antidepressants immediately as her mood had improved, but her GP had insisted on reducing the dose gradually:

*‘She wanted me to wind it down…she made me have one more lot’.* (70F08S2)

Thus respondents pointed to differences between themselves and their treating clinicians in perceptions of the diagnosis of depression and its management. Some managed these differences by doing what felt right, often without consulting their doctors.

Other interviewees had been taking their antidepressants but considered themselves ineligible because their depression was not severe enough to meet the inclusion criteria in the trial invitation. These respondents reported that: they were ‘not very depressed’; they were on maintenance doses of antidepressants; their antidepressants were for comorbid conditions like anxiety; their depression was not the main problem; or their mood had improved as a result of taking antidepressants:

*‘The thing was I’d been on tablets but they seemed to have worked’.* (69F14S2)

Some interviewees reported that, to be of use to the trial, they needed to be much more unwell than they were:

*‘I didn’t think you’d learn anything from me’.* (73F10S2)

Thus respondents and their clinical teams differed in their interpretation of eligibility for the trial. The fluidity of the diagnosis of depression may have allowed these differing interpretations that led to interviewees excluding themselves from the trial. The imprecision of the initial screening process via electronic health records may have given further scope for interviewees to exclude themselves and not progress to the full assessment of eligibility (by a member of the REFRAMED team). As one participant commented:

*‘Our GP practice must have sent the letter to everyone with the word “depression” in their records rather than going for the precise criteria’.* (66M12S2)

In summary the term ‘self-excluders’ describes the nine respondents who labelled themselves as ineligible after reading the initial invitation. Typically they did not deliberate on the decision, but soon returned the ‘opt-out’ form to the trial team.

#### Stage 3: assessing own need for trial therapy and potential to benefit

Other participants focused their decision making on the trial therapy. This is distinct from stage 2, in that informants who progressed to stage 3 considered the trial’s potential to benefit their health, rather than their potential to benefit the trial. They viewed the trial as offering an adjunct to current treatment, and focused their decision making on whether they were likely to benefit from the trial therapy. These seven interviewees, whom we term ‘treatment decliners’ indicated that once they had assessed that they did not need the trial therapy they decided to decline:

*‘I don’t need it. If I did need it, then yes, it’s good’.* (67F01S3)

These informants saw the trial invitation as offering help to manage their depression. Whilst they acknowledged that they were depressed, some described their depression as not as severe as others’, and therefore in less need of help:

*‘I don’t think that I’m that ill enough to warrant anything a great deal anyway, if you know what I mean. There are people far more depressed than what I am and need more help than I do’.* (67F01S3)

Other interviewees compared their present state with past episodes of depression. Several claimed they were better able to ‘cope’ with their present state than with past episodes, and therefore did not feel in need of the trial therapy:

*‘I thought, well I’m not actually, I mean, I’m bumping along on a low dose of antidepressants, I’ve retired from work, things are going reasonably’.* (66M12S2)

Despite the trial invitation stressing randomisation, all interviewees assumed they would receive the trial therapy. They made their decision by focusing on what would happen should they receive the trial therapy, rather than on the uncertainty of receiving one of two possible allocations. Some did go on to reflect on the difficult situation that could arise if a hypothetical depressed person focused their decision on their need for the treatment but was randomised to ‘usual care’. Informants emphasised how help was often lacking for people with depression and people were sometimes ‘desperate’ for treatment. In this context, one informant talked of how it was ‘*almost cruel*’ to offer people the chance to enrol into a trial but then not provide the trial treatment, and advised that people could experience feelings of frustration and rejection:

*‘People are sometimes desperate for something new or different that will get rid of the pain…for people with mental health issues where feelings of suicide pop up now and again it can be almost cruel if you were not to be chosen[…]Feelings of distress and frustration can be ever so amplified. You can feel so disheartened’.* (46F16S4)

Similarly, another interviewee who had participated in a trial of psychological therapy for depression (which he had completed not long before being invited into REFRAMED) described how he had enrolled in the previous trial because he had wanted help for his depression; and he had declined to participate in REFRAMED because his depression was much improved as a consequence of receiving the active psychological intervention in the previous trial. He recognised there was a chance he might not receive the psychological intervention in the previous trial; however he had enrolled with the clear aim of being assigned to psychological therapy:

*‘That was my target. I aimed to get the assessments right, so they would put me on the [trial therapy], because I wanted something to help me. No question of it, that was my goal. I never thought any different’.* (62M07S3)

Thus there was also a belief that the randomisation outcome depended on the baseline assessments.

This and other accounts saw trials as providing access to potentially life-prolonging and life-enhancing treatment not otherwise available. The perception was that trials are fulfilling health needs, rather than providing an impartial mode of resolving clinical uncertainty. Thus not to receive the trial treatment was problematic for people seeking novel healthcare where few other options were available. In declining REFRAMED, however, patients did not feel they ‘needed’ the trial therapy to manage their depression. However, it is clear from these accounts that, if interviewees had felt they needed the therapy, they would have considered enrolling in the trial with the aim of accessing the trial therapy to manage their depression.

#### Stage 4: deliberating burdens and benefits of trial participation

The remaining two interviewees deliberated about the costs and benefits of trial participation, but only after deciding that they could benefit from the trial therapy. We describe them as ‘trial decliners’. They considered the burden of the research procedures and the commitment required to participate. Personal circumstances, like caring and work responsibilities, were key considerations alongside the distance and time from home to therapy and other inconveniences caused by participation.

Interviewees expressed this in terms of comparing burdens and rewards. The burdens arose from the time commitment, both to therapy and research follow-up; one focused on the number and length of therapy sessions, regarded as time-consuming and ‘intense’, whilst the other focused on the follow-up period of 18 months:

*‘The long-term commitment was a nightmare for me as I was looking for work, going for interviews and not really knowing what I would be doing or where I would be over the next 18 months’.* (46F16S4)

This debate was important only to the two people who judged that they were eligible and could benefit from the trial therapy – the ‘trial decliners’. Most interviewees decided to opt out of the trial earlier in the deliberation process and did not consider inconvenience as a primary reason for not participating; for them eligibility and need for the trial therapy trumped inconvenience.

## Discussion

### Summary of main findings

The 20 interviewees had positive views of research and of the aims of the REFRAMED trial in particular. Many had experience of research participation. The interviews enabled us to identify four stages in the process of deciding whether or not to participate in the REFRAMED trial. At each stage some respondents concluded their deliberation and opted out. In stage 1 the ‘*prior decliners’* opted out, who have an established position of declining trial participation, stemming from personal circumstances, for example viewing themselves as ‘too old’. In stage 2, the ‘*self-excluders*’ who use the trial eligibility criteria to declare themselves ineligible opted out; they see their illness and its management differently from the clinical team who invited them to participate. In stage 3 the ‘*treatment decliners’* opted out, who perceive that they may be eligible, but focus on their health needs and decide that they do not need the trial therapy. In stage 4 the ‘*trial decliners’* opted out, who perceive that they may be eligible and in need of the trial therapy, but focus on the burden of trial participation and decide that that outweighs potential benefits.

### Strengths and limitations

Our study adds to the very sparse literature on non-participation in randomised trials. To our knowledge this is the first qualitative study to explore explicitly how decisions to decline invitations to mental health trials were made and to present the results in a conceptual framework of decision making.

There are gender and age differences in the presentation and diagnosis of depression [44, 45], and most primary care depression trials enrol many more females than males [46]. Our sample of 14 (70 %) women and six (30 %) men, with ages ranging from 18 to 77 years, reflects the demographics of depression trials and is a strength of this study.

We used telephone and e-mail interview methods and it is possible that, compared with face-to-face interviews, these may compromise rapport, probing and interpretation of interview responses [47]. However, using these methods enabled us to interview a hard-to-reach group who otherwise may not have engaged [48, 49] and to achieve a degree of anonymity which arguably helped interviewees to disclose their experiences.

It is possible that interviewees present themselves as rational deliberators in studies of this sort, because that is what they perceive is expected of them. We minimised this risk by asking interviewees simply to report what happened when they received the trial invitation, rather than to provide detailed elaborations of their decision making process and some – the ‘prior decliners’ who had previously made similar decisions to decline other trials – clearly reported that they made the decision with little deliberation. Some interviews occurred months after the initial refusal. While some respondents had difficulty recalling details, most recalled the invitation and decision process in detail and provided vivid accounts.

As in all studies of volunteers, informants selected themselves. However, participants represented only 16 % of decliners, which may limit the transferability of our findings. Interviewees expressed very positive views of research, presumably because, like other studies of non-participation, we could not access those averse to research. However, we doubt whether research-averse individuals could help to enhance recruitment, as they would not respond to recruitment interventions.

Patients who declined after being directly approached by clinicians to participate in REFRAMED also could not contribute to this study. Such patients may have offered different views, particularly around eligibility and self-exclusion issues, since clinicians were perhaps more likely to approach those whom they were confident would meet the trial eligibility criteria.

Whilst we undertook purposive sampling, the small numbers of patients who responded limited the scope of that. Despite this we did reach data saturation with those interviewed. Finally, the novel treatment in REFRAMED was aimed at patients with refractory depression and was particularly intense, so findings may not be transferable to other depression trials.

### Comparison with existing literature

Our meta-synthesis [23] shows that patients’ decisions to enter depression trials depend on: their health at the time of the invitation; their attitudes towards the research and trial interventions; and the demands of the trial. Our conceptual framework describes how decisions to participate require judgment between ‘risk and reward’. This qualitative study supports that meta-synthesis by showing that in making their decisions, respondents balanced their current health and whether they would benefit from the trial therapy against the burden of participating in the therapy including travel and time. In planning this study, we sought to contribute to existing knowledge. For example, we focused on patients under-represented in the previous literature by exploring how those who opted out of REFRAMED made their decisions.

Our findings reflect the wider decision-making literature, in particular the ‘deliberation and determination’ framework [25]. This framework differentiates between the pre-decisional process of deliberation, the act of determination and post-decisional outcomes. Our findings and the stages appear to match this process of ‘deliberation’, in which the person considers the invitation in light of their eligibility, experiences and need; and determination, which is the act of choosing to not participate. Our classification of individuals as ‘prior decliners’, ‘self-excluders’, ‘treatment decliners’ and ‘trial decliners’ appears to reflect the ‘determination’ phase of the deliberation and determination framework.

Our findings in this subgroup contrast with the general literature which suggests that altruism is a major reason for research participation [50–52]. Our respondents initially assessed their eligibility for the trial, then focused on their need for the trial therapy, and their potential to benefit. There is evidence that perceived ineligibility can lead people with depression to decline trial participation [24], and that patients participating in trials focus on the therapy under review and consider personal benefits from it [53–57]. The term ‘conditional altruism’ describes willingness to help others that inclines people to participate in trials, but does not clinch trial participation unless they judge that this will benefit them personally [57]. Whilst interviewees appeared to understand that randomisation meant that those who enrol might not receive the trial intervention, their accounts revealed the perception of randomisation in treatment trials as fundamentally unfair, even ‘cruel’ in cases where people may be seeking treatment through trial participation. Thus our group of decliners demonstrated similar attitudes to those who enrol to gain therapeutic benefit from trial participation. A relevant concept is the *therapeutic misconception* – a blurring of research and treatment, and thus a threat to understanding the trial and its risks [58–61]. There is some evidence that patients who decline participation often misunderstand the nature of the research [62, 63]. More pertinent to our interviewees, however, may be the concept of the *therapeutic mis-estimation*, which misunderstands the likelihood of risks and benefits rather than the general purpose of trials [64].

We found that interviewees had positive attitudes to research and the trial. This contrasts with some literature on non-participation which reports that decliners are less supportive of research [65–67]. Despite not participating, our interviewees generally did not mind being invited and felt free not to participate. There is evidence that most patients with mental health problems approve of psychiatric research [50], and that non-participation does not reflect objection to research in principle [63, 68]. Patients who opt out of trials have reported that they do not object to being asked to participate, nor do they feel any pressure to do so [69].

### Implications for recruitment practice and future research

Our findings have several implications for trial recruitment and ethical and methodological research on it. First it is important to recognise that those whom we term ‘prior decliners’ are unlikely to respond to any recruitment initiative as they have an established stance of declining all trial invitations. However, other factors leading patients to opt out of trials may be open to amelioration as they do not arise from a rejection of trials or personal stances of declining such invitations.

To improve responses to postal invitations in similar trials, the most successful interventions are likely to address patients’ assessments of their eligibility and their potential to benefit from the trial treatment, rather than reducing the burden of that treatment. Trialists can influence patients’ assessments of eligibility by exploring methods of:

- managing electronic patient records to estimate eligibility more precisely;
- influencing patients’ own assessment of eligibility and their judgments of their potential to benefit from the trial treatment; and
- drafting trial invitations, for example to minimise the risk of excluding themselves as ineligible.

The wording of invitations could be evaluated to examine the effect of conveying broader criteria on the numbers initially expressing interest, and ultimately enrolled. It is unclear whether ‘self-excluders’ make the same decisions that the trial team would, and whether the trial team would also have excluded them as not meeting the inclusion criteria. Thus trialists could evaluate a trial invitation letter which lists the precise inclusion and exclusion criteria against a comparator invitation which lists only the condition under investigation (e.g. ‘depression’), to estimate how many people initially respond in each arm, how many are excluded by the trial team and how many are ultimately enrolled. While eligibility issues are complex, there may be a case for accepting the risk of attracting more patients who turn out to be ineligible rather than being too restrictive. However, our findings caution against raising patients’ expectations in a way that would be unrealistic.

We know from our study that most patients focus on their need for the trial therapy when deciding whether to participate, whatever their final decision. Thus Miller and Brody [70] and Schlichting [71] have argued for trials to serve health needs, by abandoning the traditional commitment to clinical equipoise and conducting research ‘with therapeutic intent’. This approach replaces the ethical framework of equipoise with that of non-exploitation, so as to achieve the goals of patients, clinicians and researchers [71]. Though detailed examination of this ethical dilemma is beyond the scope of this study, trialists should know that our respondents effectively supported this radical proposal. The implication of accepting the principle of research ‘with therapeutic intent’ is that trials should aim, not only for a favourable benefit-risk ratio for society, but also to avoid an unfavourable benefit-risk ratio for each trial participant [72, 73]. Our qualitative study suggests that trialists should prospectively monitor patients’ expectations of their trials and use that to inform design and delivery. Better, patient-centred explanations of the potential benefits of trial treatments may help [74]. Engaging service users and members of the public in the design and conduct of trials alongside qualitative research may be the key to this [75]. For example, qualitative research could explore patient treatment preferences [76, 77]. Thus a priority for future research is the presentation and provision of accurate and effective trial information in which patients and the public play a seminal role [78]. Retrospective but timely feedback from patients who opt out of trials can assess the acceptability of the treatment being evaluated [24]. Early inclusion of such feedback into trial recruitment procedures can increase participation rates [79]. However, all such interventions require robust evaluation, ideally through embedded randomised trials.

## Conclusions

We have studied how patients invited into a randomised trial in mental health decided not to participate. They opted out in a sequence of four stages: first, the ‘prior decliners’ who have an established position of declining trial participation; second, the ‘self-excluders’ who judge that they are ineligible; third, the ‘treatment decliners’ who decide that they do not need the trial therapy; and finally the ‘trial decliners’ who decide that the burden of trial participation outweighs potential benefits. These findings have positive implications for improving trial recruitment, because trialists can address most of these issues.

## Additional files

Additional file 1: Summary participant information leaflet. Description of data: a copy of the trial participant information that was posted to interviewees along with the trial invitation letter. (PDF 279 kb)
Additional file 2: Interview with decliners in a depression trial - topic guide. Description of data: the topic guide used to interview participants. (DOCX 28 kb)

## Abbreviations

GP: General Practitioner
MRC: Medical Research Council
NHS: National Health Service
NIHR: National Institute for Health Research
PRIMER: Primary Care Research in Manchester Engagement Resource
REC: Research Ethics Committee
RO-DBT: Radically Open Dialectical Behavioural Therapy
SURP: Service User Research Panel
