# Supplementary material for: What can we learn from trial decliners about improving recruitment? Qualitative study
Source: Trials. 2016 Oct 12;17:494. doi: 10.1186/s13063-016-1626-4 (PMC5062905; doi:10.1186/s13063-016-1626-4)
Supplement: Additional file 1: — Summary participant information leaflet. Description of data: a copy of the trial participant information that was posted to interviewees along with the trial invitation letter. (PDF 279 kb) [file 13063_2016_1626_MOESM1_ESM.pdf]

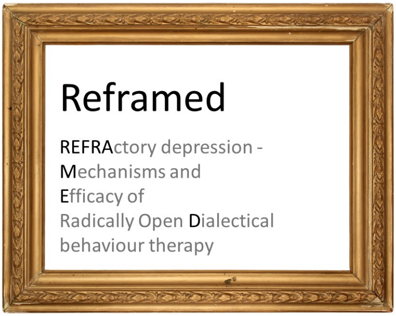

REFRAMED: REFRActory depression - Mechanisms and Efficacy of Radically Open Dialectical Behaviour Therapy

We are conducting a research study asking the question **"Does Radically Open Dialectical Behaviour Therapy reduce depression symptoms better than Standard Clinical Care such as antidepressant medication?"** and are looking for people to take part

Depression is an extremely common mental health problem that is most commonly treated with antidepressant drugs. Unfortunately, some people continue to feel depressed even though they have taken antidepressants for a while.

Recently, a new type of therapy has been developed, called Radically Open Dialectical Behaviour Therapy (RO-DBT). In order to find out whether RO-DBT can reduce depression symptoms, we need to compare two approaches to treating depression by carrying out what is called a randomised controlled trial. In this study, we will compare antidepressant medication alone with antidepressant medication plus RO-DBT.

We are hoping to include 276 people in this study.

UNIVERSITY OF Southampton

Telephone: : 01202 492126
Email: reframed.dorset@dhuft.nhs.uk
Web: www.reframed.org.uk

ISRCTN85784627
South Central REC: 11/SC/0146
Version 4 Date: 09/12/14

NHS

What's the Research about?

Standard DBT has proved to be effective for borderline personality disorder or people who harmed themselves. Radically Open Dialectical Behaviour Therapy (RO-DBT), a new treatment approach with strong roots in standard DBT, has demonstrated promise for patients with difficult-to-treat depression and related overcontrolled disorders.

We want to know if RO-DBT together with antidepressant medication is better than antidepressant medication alone by asking a lot more people to take part and checking back with these people a year after they've finished their treatment to see how they are doing.

In order to work out which is the best way to help people who suffer from depression, we will organise people into two groups. One group will continue to take their antidepressants as usual (the Standard Care group) and the other group will take part in a course of RO-DBT in addition to taking their medications.

As part of the research we will ask all participants from both groups to chat, privately, with a researcher several times over the next 12, and if possible 18, months. The researcher will ask you questions about how you have been feeling and look for any signs of low mood or depression. You will never have to answer any question you don't want to.

Because we also want to know *how* this therapy might work, we will further ask people to complete a questionnaire every month during the first year, and if possible, once more 6 months later.

What is RO-DBT and what will happen if I am in the RO-DBT Group?

RO-DBT is a type of talking therapy that is based on the idea that the way people think and behave affects how they feel. During RO-DBT sessions, the patient and therapist discuss difficulties the patient is experiencing and how their thoughts and feelings affect the problem. The patient and therapist then work together to find ways of helping the person cope with their depression.

If you are in this group, you will be invited to take part in a RO-DBT programme run by a trained and closely supervised therapist. The duration of the therapy is approximately 29 weeks. The RO-DBT treatment involves 1 hour weekly individual sessions and 2.5-hour weekly group sessions. As part of this process, you may be asked to think about some of the issues discussed between sessions and you are asked to keep a diary.

What will happen if I am in the Standard Care group?

If you are in this group, we would prefer you to continue to take your prescription for the duration of the study in the way you and your GP decide is appropriate. This is currently the recommended treatment for people who suffer from depression. However, taking part in this study does not mean you would *have* to continue to take your medication; if you and your GP decided it was the right time for you to stop, we would support that decision. We will regularly ask you how you are getting on. We will also not discourage you from seeking other types of treatment, such as psychotherapy.

Who can take part?

We are looking for people who:

- ✓ Are 18 or over
- ✓ Are currently depressed
- ✓ Have been taking antidepressant medication for at least 6 weeks during their current episode. This means you do not have to be taking them at the moment, as long as you have tried them for at least 6 weeks.

Will my taking part in this study be kept confidential?

Yes, all information collected about you during the course of the research will be kept confidential in line with the normal NHS and clinical research policies.

How do I find out more?

This is a very short summary about the study, if you would like to find out more then you can do so by

- returning the enclosed ready prepared letter,
- telephoning 01202 492126 ;
- or emailing reframed.dorset@dhuft.nhs.uk

Someone working on the study will then send you more information about this study and arrange a time to meet you to answer any questions that you may have.

Thank you for reading this and for considering taking part in this study.

www.REFRAMED.org.uk
