# Supplementary material for: What can we learn from trial decliners about improving recruitment? Qualitative study
Source: Trials. 2016 Oct 12;17:494. doi: 10.1186/s13063-016-1626-4 (PMC5062905; doi:10.1186/s13063-016-1626-4)
Supplement: Additional file 2: — Interview with decliners in a depression trial - topic guide. Description of data: the topic guide used to interview participants. (DOCX 28 kb) [file 13063_2016_1626_MOESM2_ESM.docx]

**Additional file 2**

**Interview topic guide**

**The beginning of the interview covered introducing the researcher and research, agreeing appropriate time for the interview, obtaining consent for the interview, to audio record the interview and confidentiality.**

**Questions**

1. Firstly, you’ve been kind enough to agree to be interviewed about not taking part in the REFRAMED trial. I’m interested in exploring a bit about what interested you to agree to be interviewed?
2. Do you have any experience of taking part in research before? *If yes, PROBE*
3. I understand some time has passed since you were sent the letter about the research REFRAMED study. If I can ask you to think about the study, do you remember what REFRAMED is?
4. If you can think back about the REFRAMED study, what do you remember about your experience of being asked to take part in the REFRAMED study?

**PROBES**

- 1. From the very beginning of the letter dropping through your letterbox, can you talk me through what happened?
  2. Is there anything that comes to mind about it?

1. I’m really interested in working out what the process is, when someone is making a decision about whether or not to take part in a study. We don’t know about HOW people go about making the decision about whether they want to take part in research, so to get an insight into this from you, I’m going to ask a few probing questions:

**PROBES**

- 1. What did you think when you first saw the letter?
  2. Did you open the letter immediately, or did you wait a while?
  3. After you opened the letter, did you read the information leaflet straight away?
  4. What thoughts went through your mind?
  5. Did you weigh up any positives or negatives about taking part?
  6. Did you discuss it with anyone else?
  7. Did you feel conflicted at all?
  8. Did you feel you would be eligible to take part?
  9. How did you feel about being contacted?
  10. How did you feel about being sent a letter through your clinical team? Would other forms of communication have been better?
      1. – Say, by email, or a phone call, or someone approaching you in person, or your GP/nurse or other clinician telling you about the study?

1. As far as possible, did you get a chance to read the information leaflet?
2. Have you heard about a randomised controlled trial before?
3. Can you tell me a bit about what a randomised controlled trial means to you?
4. Can you tell me how you *feel* about the possibility of taking part in a research study?
5. How would you feel if you knew that patients like you had helped to design this study?

(***If not already known***)

1. Would you say that your reasons for not wanting to take part only apply to this particular research study? If so, could you please tell me why?
2. What would have had to change for someone like you to feel it more worthwhile to take up the offer to part in the study?
   - 1. What would have had to change in the study?
     2. The study treatments? The location? The amount of time involved?
3. What type of research study might you be interested in taking part in?

***(If they read the information sheet)***

“To remind you, the information sheet says that people taking part in the research trial will be randomly placed into one of two groups. One group would receive Dialectical Behavioural Therapy, which includes one to one sessions with a therapist as well as taking antidepressants, plus group therapy. The other group would not receive Dialectical Behavioural Therapy and would instead aim to remain on their antidepressants during the study.”

***If applicable***

1. When you read the information, did you have a strong idea about which group in the research study you would like to be placed in, if you were considering taking part in it?
2. How strong was your preference?
3. How did you feel about the idea of being *randomly* placed in to either the group who received Dialectical Behavioural Therapy, or the group who aimed to remain on their antidepressants?
4. Could you please tell me how you felt about the possibility of remaining on your antidepressant medication as a way of treating depression?”
5. How do you feel about psychological therapy, compared to antidepressants, for treating depression?”

***(If applicable)***

The summary pamphlet also briefly described what is involved in taking part in Dialectical Behavioural Therapy.

1. ***(If applicable)*** When you read the information, did you think that Dialectical Behavioural Therapy may be suitable for you?
2. “Those are all of the questions that I have. Is there anything else that you would like to add about your reasons for not wanting to take part in this research study?”

**------End by thanking participant-----**
